# Fertility management and outcomes after CAR T-cell therapy: an international survey from the Cellular Therapy and Immunobiology working party of the European Society for Blood and Marrow Transplantation

**DOI:** 10.1016/j.eclinm.2026.104014

**Published:** 2026-06-11

**Authors:** Giorgio Orofino, Isabel Sánchez-Ortega, Juana Schwartz Mota, Andrea Aroldi, Cristina Castilla-Llorente, Camille Bigenwald, Jean Hugues Dalle, Karima Yacouben, Roberta Di Blasi, Eugenio Galli, Victoria Grandage, Andrea Palasciano, Larry Bacon, Georg-Nikolaus Franke, David Fandrei, Maximilian Merz, Lindsay George, Lucía Lopez-Corral, Judith S. Hecker, Daniele Mannina, Stefania Bramanti, Juan Alberto Martin Gonzalez, Núria Martínez-Cibrian, Soeren Lykke Petersen, Kamila Polgarova, Rachel Protheroe, Deborah Richardson, Ron Ram, Shlomo Elias, Bastian von Tresckow, Friedrich Stölzel, Iwona Streiss, Nicolas Vallet, Jeroen Knippenberg, Ana Alarcon Tomas, Simona Pagliuca, Florent Malard, Jürgen Kuball, Annalisa Ruggeri

**Affiliations:** a IRCCS San Raffaele Scientific Institute, Milan, Italy; b EBMT Executive Office, Barcelona, Spain; c EBMT Leiden Study Unit, Leiden, Netherlands; d Hematology Division, Fondazione IRCCS San Gerardo dei Tintori, Monza, Italy; e Gustave Roussy Cancer Campus, Villejuif, France; f Robert-Debré Academic Hospital, Groupe Hospitalier Universitaire Assistance Publique-Hôpitaux de Paris Nord, Université Paris Cité, Paris, France; g University of Paris APHP, Saint-Louis Hospital, Hemato-oncology, DMU DHI, Paris, France; h Fondazione Policlinico Universitario Agostino Gemelli IRCCS, Rome, Italy; i University College London Hospital, London, UK; j Hope Directorate - St. James's Hospital, Dublin, Ireland; k University of Leipzig 1, Leipzig, Germany; l Birmingham Centre for Cellular Therapy and Transplant (BCCTT), Birmingham, UK; m Hospital Clinico, Salamanca, Spain; n Technical University of Munich, School of Medicine and Health, Center for Translational Cancer Research (TranslaTUM), Munich, Germany; o IRCCS Istituto Clinico Humanitas, Rozzano, Italy; p Hospital Univesitario Virgen del Rocío, Seville, Spain; q Hospital Clinic, Barcelona, Spain; r Copenhagen University Hospital, Rigshospitalet, Copenhagen, Denmark; s General Faculty Hospital, 1st medical Faculty, Charles University, Prague, Czech Republic; t University Hospitals Bristol and Weston NHSFT, Bristol, UK; u Southampton General Hospital, Southampton, UK; v Bone Marrow Transplantation Unit,Tel Aviv Sourasky Medical Center and Faculty of Medicine, Tel Aviv University, Tel Aviv, Israel; w Department of Bone Marrow Transplantation and Cancer Immunotherapy, Hadassah Medical Center, Faculty of Medicine, Hebrew University of Jerusalem, Jerusalem, Israel; x Department of Hematology and Stem Cell Transplantation, West German Cancer Center and German Cancer Consortium (DKTK Partner Site Essen), University Hospital Essen, University of Duisburg-Essen, Essen, Germany; y Division of Stem Cell Transplantation and Cellular Immunotherapies, University Hospital Schleswig-Holstein, Campus Kiel, Kiel, Germany; z National Research Institute of Oncology, Warsaw, Poland; aa Hematology and Cell Therapy Department, Inserm U1069, Tours University Hospital, Tours, France; ab Hospital Universitario Puerta de Hierro, Madrid, Spain; ac CHRU NANCY, Vandoeuvre les Nancy, France; ad Service d’Hématologie Clinique et de Thérapie Cellulaire, Hôpital Saint Antoine, AP-HP, Sorbonne Université, Centre de Recherche Saint-Antoine INSERM UMRs938, Paris, France; ae University Medical Center Utrecht, Utrecht, Netherlands

**Keywords:** CAR-T, Fertility preservation, Reproductive outcomes, Pregnancies

## Abstract

**Background:**

CAR T-cell therapy has become a highly effective treatment for hematological malignancies, and emerging evidence indicates promising benefits for non-oncohematological conditions. As its clinical use broadens, understanding long-term outcomes and late complications is crucial. One critical yet understudied area is fertility, for which current evidence remains limited and no formal guidelines provide direction for patients undergoing CAR T-cell therapy.

**Methods:**

To address this gap, we conducted a cross-sectional survey on behalf of the Cellular Therapy and Immunobiology Working Party (CTIWP) of the European Society for Blood and Marrow Transplantation (EBMT) focusing on current practices, existing challenges, and reported reproductive outcomes. Questionnaires were distributed electronically (via SurveyMonkey) between Jan 8, 2025 and April 18, 2025 to 247 EBMT-affiliated centers assessing current fertility-related practices and procedures around CAR T-cell therapy. A second, complementary questionnaire was circulated between Dec 23, 2025 and April 9, 2026 to gather detailed information on reported pregnancies following CAR T treatment.

**Findings:**

99 of 247 (40%) centers answered and were included in the analysis. At data censoring, 24 pregnancies were reported in 19 patients, resulting in 18 live births, 2 ongoing pregnancy (one with twins), and 4 miscarriages. Eighteen pregnancies occurred in female CAR T-cell recipients, and six were reported by male recipients through their partners. In patients achieving pregnancy, B cell lymphoma was the most common indication for treatment. Pregnancies in the female cohort occurred naturally in 83% of cases (15/18). Among patients with data, the median time between CAR T-cell infusion and delivery or miscarriage was 3 years (range 4 months–6 years). Although both low- and high-grade Cytokine Release Syndrome (CRS) and Immune Effector Cell–Associated Neurotoxicity Syndrome (ICANS) were reported among these patients, these events did not appear to influence pregnancy outcomes, acknowledging the small sample size. While most centers (52/63, 83%) reported offering fertility counselling before CAR T-cell infusion, 11 centers (17%) indicated that they do not routinely inform patients of the potential reproductive risks. Most centers (79%) offered fertility preservation procedures to male and female patients. The most common barriers to fertility preservation referral were the urgency of initiating bridging therapy to CAR T-cell infusions due to active or rapidly progressive disease in aggressive disease and extensive prior chemotherapy exposure, defined as more than three previous treatment lines. For female patients, the predominant approaches were oocyte cryopreservation (63%) and ovarian tissue cryopreservation (59%). Among male patients, semen collection and cryopreservation was the most frequently used method (93%). Endocrinologic follow-up practices after CAR T-cell therapy varied substantially across centers.

**Interpretation:**

We report the largest series of pregnancies and live births after CAR T in patients with hematological malignancies and autoimmune diseases, and the first within Europe. As CAR T-cell therapy is increasingly administered earlier in the treatment algorithms and to younger populations, integrating standardized fertility counselling and preservation strategies into routine care will be essential. The reproductive success highlights the urgent need for robust research and formalized guidelines in this evolving field.

**Funding:**

None.

**Research in context**

**Evidence before this study**

To evaluate the current state of knowledge regarding fertility outcomes and procedures in patients treated with CAR T-cell therapy, we conducted a literature search of the PubMed database up to January 2026 using the terms “fertility” OR “pregnancy” AND “CAR-T” AND “cellular therapy.” This search yielded 19 publications. Among these, one study reported the results of a survey conducted across CIBMTR centers in 2022, focusing on fertility-related practices and outcomes in the context of cellular therapies, with few cases of successful pregnancies. A further twelve publications were narrative or systematic reviews addressing the impact of chemotherapy and immunotherapy on fertility outcomes in adult and paediatric oncology populations. Three publications were isolated case reports describing successful pregnancies following CAR T-cell therapy outside Europe. The remaining three studies were not directly relevant to the objectives of this review and were therefore excluded.

**Added value of this study**

In this multicenter EBMT survey, we report the largest series to date of pregnancies following CAR T-cell therapy, and the first within European centers. We describe 23 pregnancies in 18 patients, resulting in 18 live births, one ongoing pregnancy, and four miscarriages at the time of data censoring. Pregnancies occurred both in female CAR T-cell recipients and in partners of male recipients, with most achieved through natural conception (82% of cases in the female patients).

These findings provide previously unavailable and reassuring evidence regarding reproductive outcomes after CAR T-cell therapy. In addition, our study offers a comprehensive overview of fertility counselling, preservation strategies, and post-therapy follow-up practices across participating centers. We reveal substantial heterogeneity in clinical approaches and identify key unmet needs, including the lack of standardized fertility counselling, limited access to fertility preservation options, and insufficient psychosocial and endocrinologic follow-up.

**Implications of all the available evidence**

Taken together with available evidence, this multicenter EBMT survey addresses a timely and clinically important gap in survivorship care after CAR T-cell therapy. Whereas prior data were limited to small series and isolated case reports, our findings provide the most comprehensive evidence to date that pregnancy is feasible after CAR T-cell therapy, both in female recipients and in partners of male recipients, most often through natural conception. These results support the integration of fertility considerations into routine clinical care for patients undergoing CAR T-cell therapy and provide instrumental information to clinicians to guide the patients in their long term life expectancy after a diagnosis requiring chemotherapy and cell therapy. Nevertheless, the substantial heterogeneity observed in fertility counselling, preservation strategies, and post-treatment follow-up highlights persistent unmet needs. This variability underscores the need for standardized, multidisciplinary approaches, improved access to fertility preservation, and systematic long-term psychosocial and endocrinologic follow-up to optimize survivorship outcomes.

## Introduction

Chimeric antigen receptor (CAR) T-cell therapy has demonstrated high and durable remission rates in both clinical trials and real-world settings among patients with B-cell and plasma cell malignancies.^1^, ^2^, ^3^, ^4^, ^5^ Several CAR T-cell products are now approved by the U.S. Food and Drug Administration for the treatment of hematological malignancies, with emerging evidence of clinical activity in solid tumors, particularly within the pediatric population.^6^^,^^7^

According to data reported by the Center for International Blood and Marrow Transplant Research (CIBMTR) and the European Society for Blood and Marrow Transplantation (EBMT), more than 20,000 patients have been treated worldwide with CAR T-cell therapies to date.^8^^,^^9^ This number is expected to increase substantially in the coming years. As of 2025, more than 16,000 CAR T cell treatments have been captured only in the EBMT Registry (https://www.ebmt.org/registry/ebmt-car-t-data-collection-initiative), a figure that likely underestimates the true number of cases in Europe. This growth is driven by the expanding number of approved indications, as well as ongoing registration studies and clinical trials in both malignant and non-malignant diseases.

A number of analyses focused on short term complications, in particular Cytokine Release Syndrome (CRS) and Immune Effector Cell–Associated Neurotoxicity Syndrome (ICANS), showing variable ranges of these manifestations impacting on mortality in the early CAR T period,^10^ but the potential long-term effect are poorly reported. With the rapid expansion of CAR T-cell therapy, increasing attention has been directed toward the evaluation of late effects and complications. Among these, the potential impact on fertility is of relevance, as a significant proportion of treated patients are young individuals of reproductive age. In particular, exposure to chemotherapies cycles before CAR-T, but also alkylating agents within standard lymphodepleting regimens may significantly contribute to gonadotoxicity. Indeed, these agents are well known for their detrimental effects on gonadal function, potentially impairing fertility.

This issue becomes even more critical as CAR T-cell therapy is increasingly considered for autoimmune diseases, a population in which patients are often young and pregnancy is frequently challenging due to disease-related factors and prior or ongoing immunosuppressive treatments.^11^^,^^12^ In this context, demonstrating the absence of significant long-term impairment of fertility following CAR T-cell therapy could have important therapeutic implications.

In 2022, the CIBMTR reported the results of a survey revealing substantial heterogeneity and limited standardization in fertility preservation counseling and practices for patients undergoing CAR T-cell therapy.^13^ That survey also documented a small number of successful pregnancies following treatment (7 pregnancies with 5 live births). More recently, isolated case reports describing spontaneous pregnancies in patients treated outside Europe without assisted reproductive techniques after CAR T-cell therapy have further underscored the need for systematic and standardized research in this area.^14^^,^^15^

For these reasons, we present an updated overview of fertility-related considerations following CAR T-cell therapy across EBMT centers, with a specific focus on current practices, existing challenges, and reported reproductive outcomes.

## Methods

### Study design and ethics

We performed a cross-sectional survey on behalf of the Cellular Therapy and Immunobiology Working Party (CTIWP) of the EBMT after approval by the institutional review board of the CTIWP (reference number: CTIWP-8420045). Data were collected according to EBMT rules. Patients or legal guardians provided written informed consent for data collection and analysis in accordance with the Declaration of Helsinki and with the centers’ ethical research guidelines. Sex was recorded from medical records and reported as a biological variable.

### Survey development

The survey questionnaire was developed and reviewed by an interdisciplinary team of two statisticians and six experts in hematopoietic cell transplantation (HCT) and cellular therapies. The questionnaire was validated by two gynecologist specialists. Items were grouped into domains covering demographics; CAR T-cell therapy and fertility support, preservation, and procedures; preservation procedures; pregnancy and success of pregnancy outcome; and fertility procedures, with predominantly structured response options supplemented by Likert-scale and free-text responses. The full survey questionnaire is available in the Supplementary Materials (Supplementary Table S1).

Centers were tracked via EBMT Center Identification Codes. Respondents were instructed to respond on behalf of their center (one survey per center) and to consider all relevant cases, including female patients who became pregnant and male patients whose partners became pregnant. Centers with no experience with fertility and fertility preservation procedures were encouraged to provide this information in the survey.

The primary aim of the survey was to map the clinical practices related to fertility counselling and preservation among centers performing CAR T-cell therapies. In addition, the survey sought to assess the impact of CAR T-cell therapy on fertility, reproductive health, and gonadal function, including the number of patients who conceived or fathered children after CAR T-cell treatment.

### Survey distribution

The survey was distributed electronically between Jan 8 and April 18 2025 to 247 EBMT affiliated centers and was addressed to cellular therapy physicians assessing current fertility-related practices around CAR T-cell therapy. The questionnaire was distributed to participating centers using the SurveyMonkey platform. Demographic characteristics of patients were assessed within this survey. A second, complementary questionnaire was then circulated between Dec 23, 2025 and April 9, 2026 to gather detailed information on reported pregnancies following CAR T treatment. The second questionnaire was sent to either centers that completed the first survey or did not.

### Statistical analysis

Data from the responding centers were analyzed using descriptive and univariate statistical methods using SPSS (version 31.0.1.0 IBM). Missing responses were excluded from the analyses. Results were reported as percentages of centers that provided valid responses to each item. Free-text responses were not included in the statistical analysis due to their heterogeneous nature. Subgroup analysis by patient sex was performed for reported pregnancies and described descriptively. No sensitivity analysis was performed, as the study's descriptive design did not require it.

### Role of the funding source

No funding was received for this work.

## Results

### Demographic characteristics of responding centers

**Fig. 1:**
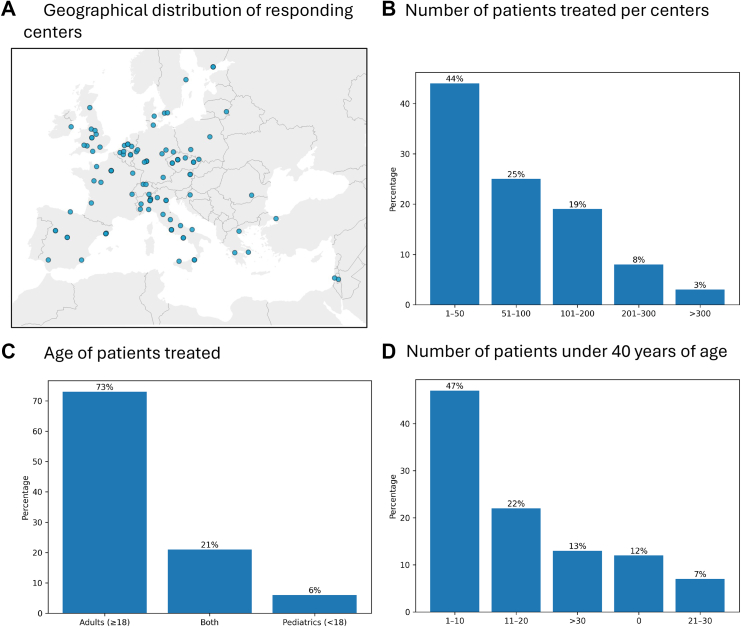
**Demographic characteristics of responding centers.** (A) Geographic distribution of participating CAR-T centers across Europe. (B) Total number of patients treated with CAR-T cell therapy per center. (C) Age groups of patients treated with CAR-T cell therapy at participating centers. (D) Number of patients under 40 years of age treated per center.

Overall, 99/247 centers (40%) responded to the survey and/or the second questionnaire, spanning 21 countries (Fig. 1A). In particular, 63 centers completed the initial survey addressing demographic characteristics and procedural aspects. Further 36 centers, completed the second questionnaire focusing on pregnancy outcomes.

For centers who responded to the survey (n = 63, 25%), the majority started CAR T-cell program in 2019 (range: 2017–2024), mainly using commercially available CAR T. The median number of patients treated per center was 57 (IQR, 31–118). Overall, responding centers reported a wide variability in experience with CAR T-cell therapy. Most centers (n = 28/63, 44%) treated ≤50 patients, while 16/63 centers, (25%) reported between 51 and 100 patients and 12/63, (19%) between 101 and 200 patients. Only 5/63 (8%) centers reported higher volumes, treating 101–200 patients and 2/63 (3%) treating >300 patients (Fig. 1B).

Most centers (n = 46/63, 73%) reported to treat only adult patients, 13/63 (21%) treated both pediatric and adult populations, while 4/63 (6%) treated only pediatric patients (Fig. 1C). Regarding younger adult patients, most centers treated patients under 40 years of age, with 28/63 (47%) reporting treatment of 1–10 patients and 13/63 (22%) treating 11–20 patients. Fewer centers treated 21–30 patients (n = 4/63, 6%) or more than 30 patients (n = 8/63, 13%), while a minority reported no patients in this age group (Fig. 1D). The median number of patients under 40 years of age treated per center was 8 (IQR, 3–20).

### Pregnancy outcomes

A total of 24 pregnancies were reported in 19 patients across 13 centers (Table 1 and Supplementary Table S2). At the time of data analysis, these pregnancies resulted in 18 live births, 2 ongoing pregnancy, and 4 miscarriages. Eighteen pregnancies occurred in 13 female recipients of CAR T-cell therapy. Among these women, three experienced a spontaneous miscarriage and one underwent an elective abortion. Notably, the three patients with spontaneous miscarriage subsequently conceived again (one twice after the miscarriage), and all three later achieved successful pregnancies resulting in live births. In the female cohort, the majority of pregnancies (15 of 18 pregnancies, 83%) occurred spontaneously.

**Table 1:** Clinical characteristics of the patients achieving pregnancies after CAR T-Cell therapy.

CAR: chimeric antigen receptor, HCT: hematopoietic cell transplantation, F: female, M: male, auto: autologous, CRS: cytokine release syndrome, ICANS: immune effector associated neurotoxicity syndrome, B- ALL: B-cell Acute Lymphoblastic Leukaemia, DLBCL: Diffuse large B-cell Lymphoma, FL: Follicular Lymphoma, MM: multiple myeloma, PMBCL: Primary Mediastinal B-cell Lymphoma, CNS: central nervous system, NA: not applicable.

Six male CAR T-cell recipients reported pregnancies resulting in live births in their partners. Of these, 2 pregnancies were achieved through natural conception, while 4 required assisted reproductive techniques: three using cryopreserved semen and one using donor-derived semen.

The most common diagnosis in patients achieving pregnancy was B-cell lymphoma (Diffuse Large B-Cell Lymphoma in 7 patients, Primary Mediastinal B-Cell Lymphoma in 3 patients, Follicular Lymphoma in 2 patients and 1 patient with primary Central Nervous System Lymphoma), followed by 4 cases of B-ALL, one patient with Multiple Myeloma and one patient with Autoimmune Disease (Necrotizing Autoimmune Myopathy) (Table 1).

The median age of patients at the time of CAR-T cells was 28.5 years (range 11 years–46 years) and at the time of pregnancy was 32 years (range 17 years–47 years). The CAR-T cells were administered from 2017 to 2024, with the majority administered (14/19) before 2022.

According to the original diagnosis, for the 13 patients treated for B-Cell NHL, the median number of lines before CAR T was 3 (range 1–6), and 3 patients had received a previous autologous HCT. Two of the 4 patients treated for B-ALL had previously received an allogenic HCT and received CAR T after a median number of 3 lines of therapy (range 2–5). The patient treated for Multiple Myeloma received CART as second line after failing a previous autologous HCT. Among the 6 patients receiving an allogeneic or autologous HCT, 5 patients achieved pregnancies with assisted reproductive techniques. Most of the patients were treated with commercially available products incorporating either CD28 or 4-1BB as the co-stimulatory domain. Standard lymphodepleting regimens were used. At the time of pregnancies, patients reported either persistent or resolved B-cell aplasia, although this information was not available for all cases (information available in 15/19 patients).

The median interval between CAR T-cell infusion and conception (delivery or miscarriage) was 3 years, with a range from 4 months to 6 years. Both low- and high-grade CRS and ICANS were observed among patients who achieved pregnancies, in both male and female populations. In particular, among the three female patients who experienced a spontaneous miscarriage followed by a subsequent live birth, two had low-grade CRS and ICANS, while one experienced high-grade CRS and ICANS (Table 1).

### Access to fertility support

**Fig. 2:**
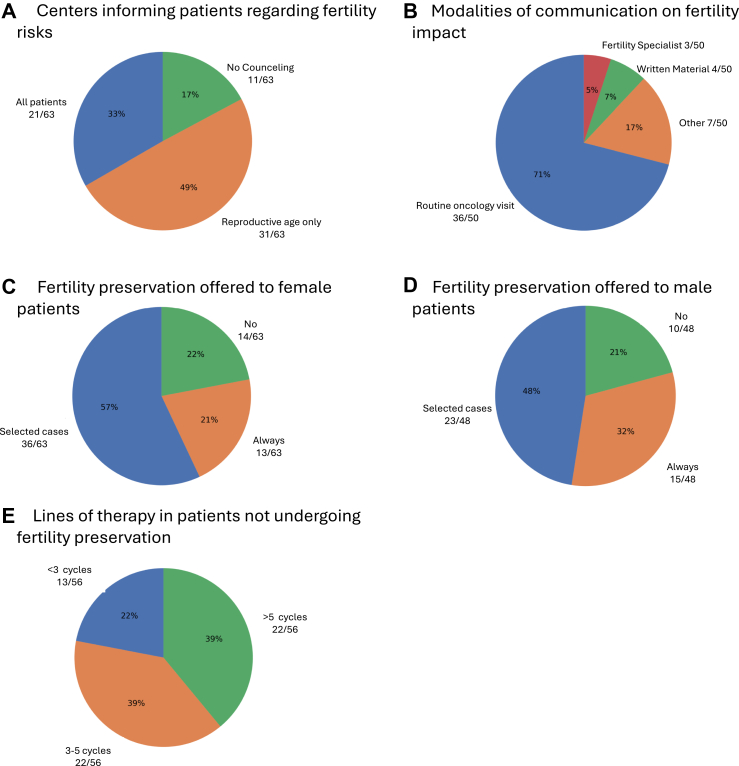
**Fertility counseling, preservation and supportive care practices in CAR-T centers.** (A) Provision of fertility counseling prior to CAR-T cell therapy. (B) Modalities of information sharing regarding fertility risks and preservation options. (C) Availability of fertility preservation procedures for female patients. (D) Availability of fertility preservation procedures for male patients. (E) Number of prior lines of therapy in patients who did not undergo fertility preservation due to previous intensive chemotherapy.

Among centers responding to the first part of the survey, most centers (52/63, 83%) reported routinely address fertility-related issues prior to CAR T-cell therapy, in particular at the moment of diagnosis, especially when induction can be delayed. Specifically, 33% of centers (21/63 centers) stated that they inform all patients about the potential impact of CAR T-cell therapy on fertility, while 49% (31/63) provide this information only to patients of reproductive age. In contrast, 17% (11/63 centers) of centers reported that fertility-related information was not routinely discussed (Fig. 2A).

Information on fertility risks and preservation options was most shared during routine oncology consultations (71%, 36/50 responding centers). Less frequently, information was provided through formal consultations with fertility specialists (5%, 3/50 centers) or via written informational materials (7%, 4/50 centers), or other modalities (17%, 7/50 centers) (Fig. 2B).

Most centers (79%) offered fertility preservation procedures to male and female patients. Fertility preservation options for female patients were offered on a case-by-case basis in 57% of centers (36/63), while 21% (13/63) reported systematically offering fertility preservation to all eligible female patients; the remaining 22% (14/63) did not provide fertility preservation procedures for female patients (Fig. 2C). Similarly, for male patients, fertility preservation was offered selectively in 48% of centers (23/48) and routinely in 32% (15/48), while 21% (10/48) reported not offering these procedures (Fig. 2D).

Access to specialized consultations was widely available, with 65% of centers (33/51) reporting the availability of a dedicated multidisciplinary team including gynaecologists, endocrinologists, or fertility specialists. However, 27% (14/51) offered such counselling only in selected cases, and 8% (4/51) reported no access to specialized fertility consultations. Despite the availability of fertility preservation programs, the majority of centers (66%, 40/60) reported that no patients had undergone fertility preservation procedures, while 25% (15/60) reported 1–5 patients, and only a small proportion (5%, 3/60) reported more than 5 patients undergoing such procedures.

Fertility preservation procedures were performed within the treating centers in 52% of cases (33/63), while 38% (24/63) of centers referred patients to external clinics, and 10% (6/63) reported a combination of internal and external services. Sixty percent of centers (26/44) considered age younger than 40 years as a referral criterion, and 67% (29/44) considered patient willingness to conceive, while other criteria were reported by 25% of centers (11/44). Nearly half of the centers (48%, 30/63) recommended fertility preservation as soon as possible, and 37% (23/63) recommended it before initiation of the first chemotherapy cycle.

The most commonly reported reasons for not proceeding with fertility preservation included prior intensive or multiple chemotherapy regimens, lack of time due to active or rapidly progressive disease needing bridging therapy, postmenopausal status or advanced age, and patient or family refusal. Other cited reasons included lack of desire for future children and previous fertility preservation procedures. Financial barriers were rarely reported as a limiting factor.

Among patients who did not undergo fertility preservation due to prior intensive chemotherapy exposure, most had received three or more prior treatment lines, with 39% (22/56) having received three to five cycles and 39% (22/56) more than five cycles (Fig. 2E).

### Fertility preservation procedures and follow up

Table 2 reports the most common fertility preservation practices observed across centers. Among female fertility preservation strategies, oocyte cryopreservation and ovarian tissue cryopreservation were reported by 63% (40/63) and 59% (37/63) of centers, respectively. However, routine implementation of these procedures was uncommon, with most centers offering them only in selected cases. Overall, 70% (44/63) of centers indicated that no patients had undergone any fertility preservation procedure during the study period, underscoring limited real-world uptake despite procedural availability.

**Table 2:** Key fertility preservation and follow-up practices in CAR-T centers in female and male patients.

	Key findings
Female patients	
Cryopreservation procedures	Oocyte cryopreservation and ovarian tissue cryopreservation are available in a substantial proportion of centers but are mostly offered on a selective rather than routine basis.
Follow-up and monitoring	- Follow-up with fertility specialists after fertility preservation is commonly offered, though not uniformly across centers. - Hormonal monitoring is variable; gonadotropins (FSH and LH) are the most commonly assessed fertility markers.
Ovarian functional rest	Ovarian functional rest during CAR-T therapy is infrequently recommended; when used, gonadotropin-releasing hormone (GnRH) agonists are the preferred approach.
Male patients	
Fertility preservation procedures	- Natural semen collection and cryopreservation is the most commonly available fertility preservation technique. - Advanced procedures (testicular sperm extraction and testicular tissue cryopreservation) are available in a minority of centers and are generally offered selectively rather than routinely.
Follow-up and monitoring	Testosterone is the most frequently monitored fertility-related biomarker in male patients, while other hormonal markers are assessed less consistently.

Among male fertility preservation strategies, natural semen collection with cryopreservation was the most commonly available technique, reported by 93% (59/63) of centers. More specialized procedures were less frequently offered: testicular sperm extraction with cryopreservation was available in 27% (17/63) of centers, while testicular tissue cryopreservation was reported by 20% (13/63), with the majority of centers (80%, 50/63) indicating that this procedure was not offered. When available, advanced male fertility preservation techniques were generally applied selectively rather than routinely (Table 2).

Follow-up care with fertility specialists after fertility preservation was routinely offered by 61% of centers (38/63), offered selectively by 16% (10/63), and not available in 23% (15/63). Among centers monitoring fertility markers in women, follicle-stimulating hormone (FSH) and luteinizing hormone (LH) were the most frequently assessed biomarkers (92%, 58/63 and 88%, 55/63, respectively), followed by estradiol (57%, 36/63), progesterone (47%, 30/63), and anti-Müllerian hormone (AMH; 45%, 28/63) (Table 2). In male patients, testosterone was the most commonly monitored marker (93%, 59/63), whereas FSH and LH were assessed by 56% (35/63) and 54% (34/63) of centers, respectively; estradiol and inhibin B were rarely monitored (12%, 8/63 and 7%, 4/63, respectively) (Table 2).

Recommendations for ovarian functional rest during CAR-T cell therapy were uncommon. Only 9% of centers (5/57) reported always recommending this strategy, while 6% (3/57) did so often and 24% (14/57) sometimes; the majority (62%, 35/57) reported never recommending ovarian functional rest. When implemented, gonadotropin-releasing hormone (GnRH) agonists were the most frequently used approach (86%, 49/57), whereas progestins were rarely employed (19%, 11/57). The timing of ovarian functional rest varied across centers, reflecting the absence of standardized practice.

## Discussion

Our study demonstrates that reproductive potential can be preserved after CAR T-cell therapy. The occurrence of spontaneous pregnancies, including in patients previously exposed to gonadotoxic treatments, provides clinically meaningful evidence that fertility may remain intact in a subset of survivors. Chimeric antigen receptor T-cell therapy is transforming the management of adult patients with B-cell and plasma cell malignancies, with an increasing proportion of patients achieving durable, long-term remission.^16^ In parallel, the clinical use of CAR T-cell therapies has expanded to pediatric, adolescent, and young adult populations in both B-cell acute leukemias and now also in solid tumors, thereby substantially extending post-treatment life expectancy. As previously observed with cytotoxic chemotherapy and hematopoietic cell transplantation, the growing population of long-term survivors underscores the need for increased attention to quality-of-life outcomes and the systematic assessment of late and long-term treatment-related toxicities.^17^^,^^18^ Among these, reproductive health and fertility preservation represent critical concerns,^19^ particularly for younger patients treated with curative intent.^20^

Beyond oncology, CAR T-cell therapy has demonstrated promising clinical efficacy in patients with severe, refractory autoimmune conditions such as systemic lupus erythematosus and systemic sclerosis, as well as other immune-mediated disorders.^21^ These diseases often require lifelong immunosuppressive therapies that are associated with significant cumulative toxicities and adverse effects also on fertility and pregnancy outcomes.^22^ In these predominantly young patient populations, achieving and maintaining pregnancy is frequently challenging and may be complicated by treatment-related gonad-toxicity,^22^ disease activity,^23^ and an increased risk of disease recrudescence during the peripartum period.^24^ In this context, evidence suggesting a limited long-term impact of CAR T-cell therapy on fertility could further support its therapeutic role in autoimmune diseases, potentially favoring this treatment option for patients with reproductive aspirations.

Our findings are particularly relevant given the intensive treatment histories typical of patients undergoing CAR T-cell therapy and contribute important real-world data to a field where long-term reproductive outcomes remain insufficiently characterized.

In parallel, this study underscores the increasing integration of fertility preservation strategies into the CAR T-cell treatment pathway. Most patients had access to fertility preservation prior to lymphodepleting chemotherapy and CAR T-cell infusion, reflecting growing awareness among clinicians of the reproductive implications of these therapies.

However, the substantial heterogeneity in the information provided and in counselling practices highlights persistent gaps in standardized reproductive care. Collectively, these findings emphasize both the feasibility of achieving parenthood after CAR T-cell therapy and the urgent need for structured, equitable, and multidisciplinary onco-fertility counselling to ensure that all eligible patients are adequately informed and supported in their reproductive choices.

In particular, in the present study, we provide an updated clinical overview of fertility-related practices and reproductive outcomes following CAR T-cell therapy across participating centers. Our findings indicate that, limited progress has been made toward the standardization of fertility counselling and preservation strategies.^13^ Indeed, current approaches remain highly heterogeneous, and formal guidelines are still lacking. Variability in the timing and implementation of fertility counseling, driven by differences in local regulations and healthcare system organization, as well clinical urgent conditions, should be carefully considered when interpreting our findings, as it may significantly influence both access to and delivery of fertility preservation services across centers.

In this context, it is essential to ensure that all patients are informed about available fertility preservation options, at diagnosis for patients with hematological malignancies or prior to CAR T treatment, for those with non malignant indications. Established approaches include sperm cryopreservation in post-pubertal males and oocyte or embryo cryopreservation in females, which remain the standard of care when time allows. Ovarian tissue cryopreservation represents an important alternative, particularly for prepubertal patients or in situations requiring urgent treatment initiation, and is increasingly being adopted in clinical practice. In addition, adjunctive strategies such as the use of gonadotropin-releasing hormone analogs may be considered, although their protective effect remains a subject of ongoing investigation. Expanding access to these techniques and integrating them systematically into the CAR T-cell treatment pathway will be essential to support informed reproductive decision-making in this growing patient population. It is important to emphasize that sperm (including intratesticular sperm) or mature oocyte collection should ideally be performed at the time of initial diagnosis, as these options may no longer be feasible once systemic chemotherapy has been initiated prior to CAR T-cell therapy; therefore, early referral for specialized counselling is essential to tailor the most appropriate fertility preservation strategy for each patient.

This study has some limitations, most notably its registry-based nature. In particular, it remains challenging to accurately determine the number of patients who desire pregnancy but are unable to conceive, as such data are not systematically captured across centers.

Indeed, due to the registry-based design and the lack of systematic data on patients’ reproductive intentions, the number of patients willing to conceive after oncological treatments cannot be properly determined. However, this limitation underscores one of the key issues highlighted by our analysis: the need for standardized communication regarding fertility and reproductive goals, ideally incorporating structured counselling and psychological support. An additional limitation of this study is the response rate, with approximately 40% of centers contributing data. While this may introduce a degree of selection bias, potentially favoring centers with greater awareness of fertility issues, response rate is consistent with, and not unexpected for, multicenter survey-based studies of this nature. Importantly, pregnancies following CAR T-cell therapy remain relatively rare events; as such, it is likely that centers with direct experience of such cases were more motivated to respond, thereby enhancing the relevance of the collected data. In this context, the reported pregnancies provide valuable real-world insights for the cellular therapy community, where evidence on reproductive outcomes is still limited. Due to these considerations, we acknowledge that pregnancies may be underestimated, although our findings already exceed previous reports, which were based on small cohorts and relatively short follow-up periods.

Continued and systematic collection of fertility-related procedures and reproductive outcomes following CAR T-cell therapy remains essential to fully elucidate long-term reproductive safety. In our cohort, the interval between CAR T-cell infusion and pregnancy ranged from 4 months to 6 years. At present, there are insufficient data to define an optimal or safe time frame for conception following treatments.

Moreover, as the use of CAR T-cell therapies continues to expand, particularly among younger patients, in earlier lines of therapies and in non-malignant indications, the number of individuals willing to conceive after their treatment is expected to increase substantially. This evolving landscape underscores the urgent need for the development of standardized, evidence-based guidelines addressing fertility counselling, preservation strategies, and optimal timing of conception following therapy. Consensus panel and further data from Post-Authorization Safety Study (PASS) will help in clarify the long term safety of these treatments.

To our knowledge, this study represents one of the largest reports of pregnancies following CAR T-cell therapy to date, providing valuable real-world evidence on reproductive outcomes in this rapidly expanding therapeutic field. Importantly, we document successful pregnancies and live births in patients who experienced acute CAR T-cell–related toxicities, including CRS and ICANS. These observations suggest that the occurrence of these early inflammatory and neurotoxic complications does not necessarily translate into clinically meaningful long-term impairment of reproductive function. Although the limited sample size precludes definitive conclusions, our findings contribute novel data to an area in which evidence remains scarce. Our results provide important evidence for treating physicians in counselling long term outcomes of cellular therapies and could offer to the patients a clear picture of the treatment related burden of toxicities.

In particular, we believe that our findings have direct clinical implications. The occurrence of pregnancies, predominantly through natural conception, suggests that CAR T-cell therapy itself may be associated with a relatively low gonadotoxic profile, particularly when compared with other chemotherapy based treatments. However, reproductive potential remains strongly influenced by prior therapies, especially hematopoietic cell transplantation and cumulative exposure to cytotoxic chemotherapies. In our cohort, all patients with a history of autologous or allogeneic cell transplantation required assisted reproductive techniques to achieve pregnancy, underscoring the significant impact of previous treatments on fertility outcomes. This distinction is critical in counselling patients, as fertility outcomes should be considered within the context of the entire treatment trajectory, rather than attributed to CAR T-cell therapy alone.

The pregnancies observed in this study predominantly occurred in patients who had received CAR T-cell therapy two or more years prior, reflecting the necessary follow-up time required to assess reproductive outcomes after treatment. Given that a substantial proportion of CAR T-cell therapies have been administered only in recent years—particularly in pediatric, adolescent, and young adult populations—it is likely that the current observations underestimate the true incidence of post–CAR T-cell pregnancies. As these younger cohorts age and enter their reproductive years, and as long-term follow-up data mature, an increasing number of pregnancies are expected to be reported. Ongoing surveillance and dedicated registries will therefore be essential to more accurately characterize fertility outcomes and reproductive health after CAR T-cell therapy over time.

Notably, in particular in female patients, the majority of reported pregnancies occurred through natural conception rather than assisted reproductive techniques, indicating that preserved or recovered fertility is achievable following CAR T-cell therapy, even though the potential contribution of lymphodepleting regimens to gonadotoxicity should be considered, particularly in relation to the cyclophosphamide-equivalent dose administered, which may influence fertility outcomes in this patient population. Finally, this observation further supports the hypothesis that CAR T-cell treatment may have a limited gonadotoxic profile compared with other intensive oncologic therapies, while emphasizing the need for larger studies and follow-up to better define fertility trajectories and reproductive safety in both malignant and non-malignant indications.

## Contributors

GO, JK and AR designed the study, GO and AR wrote the manuscript, ISO, JSM helped with the data collection and data interpretation. GO, JSM, AAT, SP, FM, JK, AR prepared and reviewed the two questionnaires; AA, CCL, CB, JHD, KY, RDB, EG, VG, AP, LB, GNF, DF, MM, LG, LLC, JSH, DM, SB, JAMG, NMC, MR, SLP, KP, RP, DR, RR, SE, BVT, FS, IS, NV provided cases for the study and reviewed the accuracy of drug dosages, where applicable, for patients with reported pregnancies. JKN helped with data collection, AAT, SP, FM reviewed the study proposal. GO, ISO, JSM and AR accessed and verified the underlying data. All the authors reviewed and approved the final draft.

## Data sharing statement

The data that support the findings of this study are not publicly available due to privacy restrictions and the sensitivity of patient-level information. Data may be made available upon reasonable request to the corresponding author.

## Editor note

The Lancet Group takes a neutral position with respect to territorial claims in published maps and institutional affiliations.

## Declaration of interests

GO is supported by AIRC (Associazione Italiana Ricerca sul Cancro) fellowship (# 33208). RR reports Honoraria from Gilead, BMS, Novartis. JK reports research support from Miltenyi Biotech, Novartis and Gadeta and is inventor on multiple patents dealing with genetic engineering and shareholder of Gadeta Founders. RDB reports honoraria from Novartis, Kite Gilead, Abbvie, travel grants from Novartis and Kite Gilead, and partecipate to Advisory Boards of Novartis and BMS. EG reports honoraria from Kite Gilead and BMS and partecipate to Advisory Boards of Novartis. GNF reports honoraria from Sanofi, Novartis, Zentiva, travel grants from Kite Gilead, Sanofi, Zentiva and partecipate to Advisory Boards of Novartis, Incute, Zentiva. MM reports honoraria from Kite Gilead. LG partecipate to Advisory Boards of Kite Gilead and Autolus. SLP reports travel grant from Kite Gilead and partecipate to Advisory Boards of Kite Gilead. KP reports travel grant from Kite Gilead and partecipate to Advisory Boards of Kite Gilead and BMS. BVT reports consulting fees from AbbVie, Allogene, Amgen, BMS/Celgene, Cerus, Gilead Kite, Incyte, IQVIA, Janssen-Cilag, Lilly, Merck Sharp & Dohme, Miltenyi, Novartis, Noscendo, Pentixapharm, Pfizer, Pierre Fabre, Qualworld, Regeneron, Roche, Serb, Sobi and Takeda; reports honoraria from AbbVie, AstraZeneca, BMS/Celgene, Gilead Kite, Incyte, Janssen-Cilag, Lilly, Merck Sharp & Dohme, Novartis, Roche, Serb and Takeda; reports travel grants from AbbVie, AstraZeneca, Kite Gilead, Janssen-Cilag, Lilly, Merck Sharp & Dohme, Pierre Fabre, Roche, Takeda, and Novartis; and is member of steering committees of Regeneron and Takeda. AAT reports travel grants from Kite Gilead and Novartis. FM reports honoraria from Sanofi, Amgen, Novartis, BMS, Astra-Zeneca, Pfizer, Therakos, Priothera, MSD, Jazz Pharmaceutical and travel grants from Gilead and Sanofi. SP reports a grant from JANSSEN HORIZON Program. AR reports honoraria from Kite Gilead. All other authors declare no competing interests.

## Appendix A Supplementary data

Supplementary Table S2

Translated Abstracts

## References

[1] Locke F.L., Miklos D.B., Jacobson C.A. (2022). Axicabtagene ciloleucel as second-line therapy for large B-Cell lymphoma. N Engl J Med.

[2] Bishop M.R., Dickinson M., Purtill D. (2022). Second-line tisagenlecleucel or standard care in aggressive B-Cell lymphoma. N Engl J Med.

[3] Bock T.J., Colonne C.K., Fiorenza S., Turtle C.J. (2025). Outcome correlates of approved CD19-targeted CAR T cells for large B cell lymphoma. Nat Rev Clin Oncol.

[4] Sheykhhasan M., Ahmadieh-Yazdi A., Vicidomini R. (2024). CAR T therapies in multiple myeloma: unleashing the future. Cancer Gene Ther.

[5] San-Miguel J., Dhakal B., Yong K. (2023). Cilta-cel or standard care in lenalidomide-refractory multiple myeloma. N Engl J Med.

[6] Bufalo F.D., Angelis B.D., Caruana I. (2023). GD2-CART01 for relapsed or refractory high-risk neuroblastoma. N Engl J Med.

[7] Escobar G., Berger T.R., Maus M.V. (2025). CAR-T cells in solid tumors: challenges and breakthroughs. Cell Rep Med.

[8] Passweg J.R., Baldomero H., Atlija M. (2025). The 2023 EBMT report on hematopoietic cell transplantation and cellular therapies. Increased use of allogeneic HCT for myeloid malignancies and of CAR-T at the expense of autologous HCT. Bone Marrow Transplant.

[9] Spellman S.R., Xu K., Oloyede T. (2025). Current activity trends and outcomes in hematopoietic cell transplantation and cellular therapy - a report from the CIBMTR. Transplant Cell Ther.

[10] Brudno J.N., Kochenderfer J.N. (2024). Current understanding and management of CAR T cell-associated toxicities. Nat Rev Clin Oncol.

[11] Schett G., June C.H. (2024). CAR T cells in autoimmune disease: on the road to remission. Immunity.

[12] Müller F., Taubmann J., Bucci L. (2024). CD19 CAR T-Cell therapy in autoimmune disease — a case series with Follow-up. N Engl J Med.

[13] Ligon J.A., Fry A., Maher J.Y. (2022). Fertility and CAR T-cells: current practice and future directions. Transplant Cell Ther.

[14] Canty E.A., Broderick L., Flaherty D. (2025). First reported case of a spontaneous and healthy pregnancy in a woman with persistent CAR T-cells 5 years after treatment for diffuse large B-cell lymphoma. J Immunother Cancer.

[15] O’Reilly D., Jones C., Smith A. (2025). Neonatal outcomes following 2 cases of maternal CAR-T therapy for high-grade B-Cell lymphoma. Neonatology.

[16] Bishop M.R. (2024). Late complications and long-term care of adult CAR T-cell patients. Hematol Am Soc Hematol Educ Program.

[17] Norkin M., Hsu J.W., Wingard J.R. (2012). Quality of life, social challenges, and psychosocial support for long-term survivors after allogeneic hematopoietic stem-cell transplantation. Semin Hematol.

[18] Newcomb R., Johnson P.C., Cronin K. (2023). Quality of life, physical functioning, and psychological distress of older adults undergoing hematopoietic stem cell transplantation. Transplant Cell Ther.

[19] Purandare N., Ruiloba F., Nguyen-Hoang L. (2025). Cancer and fertility management: FIGO best practice advice. Int J Gynecol Obstet.

[20] Rodriguez-Wallberg K.A., Anastacio A., Vonheim E., Deen S., Malmros J., Borgström B. (2020). Fertility preservation for young adults, adolescents, and children with cancer. Ups J Med Sci.

[21] Scherlinger M., Nocturne G., Radic M. (2025). CAR T-cell therapy in autoimmune diseases: where are we and where are we going?. Lancet Rheumatol.

[22] Leroy C., Rigot J.M., Leroy M. (2015). Immunosuppressive drugs and fertility. Orphanet J Rare Dis.

[23] Sangle S.R., Vounotrypidis P., Briley A. (2015). Pregnancy outcome in patients with systemic vasculitis: a single-centre matched case-control study. Rheumatology.

[24] Eudy A., Siega-Riz A.M., Engel S.M. (2018). Effect of pregnancy on disease flares in patients with systemic lupus erythematosus. Ann Rheum Dis.

