# Supplementary material for: Fertility management and outcomes after CAR T-cell therapy: an international survey from the Cellular Therapy and Immunobiology working party of the European Society for Blood and Marrow Transplantation
Source: eClinicalMedicine. 2026 Jun 11;96:104014. doi: 10.1016/j.eclinm.2026.104014 (PMC13272563; doi:10.1016/j.eclinm.2026.104014)
Supplement: Supplementary Table S1 [file mmc1.pdf]

Survey on fertility procedures and outcomes for patients treated with CAR-T cell therapy on behalf of the EBMT CTIWP
Generalities

Welcome to the survey on fertility procedures and outcomes for patients treated with CAR-T cell therapy on behalf of the Cellular Therapy & Immunobiology Working Party (CTIWP) of the EBMT.

The aim of the study is to map the clinical practices regarding fertility and fertility preservation procedures among centers performing CAR-T cell therapies. In addition, this study will investigates the impact of CAR-T cell therapy on fertility, reproductive health, and gonadal function, and will determine the number of patients that have been conceiving or fathering children after CAR-T cell therapy.

Inclusion criteria: all EBMT centers administrating CAR-T cells.

We kindly invite you to participate in this survey which can be completed in 10-15 minutes, and contribute with your centre's experience. Please only fill one survey per transplant centre. If your center has no experience with fertility and fertility preservation procedures, we very much appreciate if you could provide us that information in this survey.

Note: Your progress on the survey will be saved if you close the window, so does not need to be completed in one sitting (requires that you complete it using the same device that you started it on).

Thank-you in advance for your participation.

*** 1. EBMT Center Identification Code (CIC):**

*** 2. Contact information* of the person completing this survey:**

***The personal data provided will be processed solely to gather information on fertility procedures and outcomes for patients treated with CAR-T cell therapy in EBMT centers and according to the General Data Protection Regulation (GDPR 2016/679). The personal data will be incorporated to a database property of EBMT. Data Subjects have the right of access, rectification, erasure, restriction, data portability and objection to the processing of his or her personal. If as a Data Subject you wish to exercise any of the rights listed above, please write to data.protection@ebmt.org. For further information please go to the Privacy Policy.**

First and last name of respondent

(Work) email

Name of the center

*** 3. Country**

*** 4. I agree to participate in this survey:**

◯ Yes

◯ No, please specify the reason for not participating (the survey will end after this)

Survey on fertility procedures and outcomes for patients treated with CAR-T cell therapy on behalf of the EBMT CTIWP
Demographics

*** 5. Does your center perform chimeric antigen receptor (CAR)-T cell therapy?**

- ◯ Yes
- ◯ No

Survey on fertility procedures and outcomes for patients treated with CAR-T cell therapy on behalf of the EBMT CTIWP
Demographics

6. In your center, CAR-T cell treatments are performed in:

- ◯ Adults (≥ 18 years)
- ◯ Pediatrics (< 18 years)
- ◯ Both adults and pediatrics

7. What year did your center started with EMA-approved CAR-T cell therapy activity?

[ ]

8. Total number of patients treated with CAR-T cells since the start of CAR-T therapy in your center

[ ]

9. How many patients treated with CAR-T cells were under 18 years of age?

[ ]

10. How many patients treated with CAR-T cells were under 40 years of age?

[ ]

11. How many of the total patients treated received a commercial CAR-T cell therapy?

[ ]

EBMT

Survey on fertility procedures and outcomes for patients treated with CAR-T cell therapy on behalf of the EBMT CTIWP

CAR-T cell therapy and fertility support, preservation and procedures

12. Do you inform patients about the potential impacts of CAR-T cell therapy on fertility before they begin treatment?

- ◯ Yes, all patients
- ◯ Yes, only patients of reproductive age
- ◯ No, information is not routinely provided

13. How is information about fertility risks and preservation options shared with patients prior to starting CAR-T cell therapy?

- ◯ In a formal consultation with a fertility specialist
- ◯ During routine oncology consultations
- ◯ Through informational brochures or written materials
- ◯ Other (please specify)

14. Is fertility preservation offered to <u>female</u> patients undergoing CAR-T cell therapies at your center?

◯ Yes, always

◯ No, we do not provide these kinds of procedures

◯ Yes, only in selected cases, please specify:

15. Is fertility preservation offered to <u>male</u> patients undergoing CAR-T cell therapies at your center?

◯ Yes, always

◯ No, we do not provide these kinds of procedures

◯ Yes, only in selected cases, please specify:

16. Does your center offer consultations with a gynecologist, endocrinologist, or fertility specialist for patients considering fertility preservation procedures?

◯ Yes, we do provide a specialized team consultation

◯ No, we do not have this possibility

◯ Only in selected cases, please specify:

17. Do you offer psychological support to patients considering fertility preservation procedures at your center?

◯ Yes, always

◯ No, we do not provide this service

◯ Yes, only in selected cases, please specify:

18. Since the start of CAR-T cell activity in your center, how many patients who received CAR-T cell therapy at your center have undergone fertility preservation procedures?
Number of patients:

19. Are fertility preservation procedures conducted within your center, or do patients need to be referred to an external clinic?

- ◯ Yes, procedures are conducted within our center
- ◯ No, patients are referred to an external clinic
- ◯ Both, some procedures are available at our center, while others require external referral

20. What are the criteria for referring a patient to a fertility preservation program at your center?
(Select all that apply)

- ☐ Young patients (age < 40 years old)
- ☐ Patients willing to conceive
- ☐ Other (please specify)

21. At what stage in the treatment process do you recommend a fertility preservation program to patients who are potentially eligible for CAR-T cell therapy?

- ◯ As soon as possible
- ◯ Before the first chemotherapy cycle
- ◯ Before starting CAR-T cell therapy
- ◯ After CAR-T cell therapy
- ◯ Other (please specify)

22. For patients who did not undergo fertility preservation procedures, could you provide the reasons for opting not to proceed? (Select all that apply)

- ☐ Pre-treated patients with > 3 cycles or high dose chemotherapy
- ☐ No time due to active/rapidly progressive disease
- ☐ Not an option at our center
- ☐ No wish for children
- ☐ Patient/family refusal
- ☐ Postmenopausal patients or old patients
- ☐ Procedural costs are charged to patients and not affordable
- ☐ Previously underwent fertility preservation procedures
- ☐ Other (please specify)

23. For patients who did <u>not</u> undergo fertility preservation procedures due to prior intensive chemotherapy, how many lines of chemotherapy had they received?

- ○ < 3 cycles
- ○ => 3 and =< 5 cycles
- ○ > 5 cycles

24. Is follow-up care with fertility specialists offered to patients after undergoing fertility preservation procedure?

- ○ Yes, always
- ○ No, we do not provide this service
- ○ Yes, only in selected cases, please specify:

Survey on fertility procedures and outcomes for patients treated with CAR-T cell therapy on behalf of the EBMT CTIWP

Preservation procedures

25. For the patients who underwent fertility preservation procedures, could you specify the procedure(s) that were carried out? (Select all that apply)

- [ ] Natural semen collection and cryopreservation
- [ ] Testicular sperm extraction and cryopreservation
- [ ] Testicular tissue cryopreservation
- [ ] Cryopreservation of ovarian tissue
- [ ] Oocyte cryopreservation
- [ ] No patients underwent fertility preservation procedures
- [ ] Other (please specify)

Survey on fertility procedures and outcomes for patients treated with CAR-T cell therapy on behalf of the EBMT CTIWP

26. Among all patients undergoing a fertility preservation procedure, how frequently is oocyte cryopreservation performed?

- ◯ Always
- ◯ Often
- ◯ Sometimes
- ◯ Never

27. Among all patients undergoing a fertility preservation procedure, how frequently is cryopreservation of ovarian tissue performed?

- ◯ Always
- ◯ Often
- ◯ Sometimes
- ◯ Never

28. Among all patients undergoing a fertility preservation procedure, how frequently is testicular tissue cryopreservation performed?

- ◯ Always
- ◯ Often
- ◯ Sometimes
- ◯ Never

29. Among all patients undergoing a fertility preservation procedure, how frequently is testicular sperm extraction and cryopreservation performed?

- ◯ Always
- ◯ Often
- ◯ Sometimes
- ◯ Never

30. Among all patients undergoing a fertility preservation procedure, how frequently is natural semen collection and cryopreservation performed?

- ◯ Always
- ◯ Often
- ◯ Sometimes
- ◯ Never

Survey on fertility procedures and outcomes for patients treated with CAR-T cell therapy on behalf of the EBMT CTIWP

Pregnancy and success of pregnancy outcome

Instruction:

This section includes questions about pregnancy and fertility outcomes for both male and female patients who have undergone CAR-T cell therapy. For ‘patients’, please consider all relevant cases, including female patients who became pregnant and male patients whose partners became pregnant.

31. In your center, how many patients have attempted to conceive or father children post-CAR-T cell therapy?

Number of female patients:

32. In your center, how many patients have attempted to conceive or father children post-CAR-T cell therapy?

Number of male patients:

33. Have any of your patients who attempted to conceive after CAR-T cell therapy successfully achieved pregnancy? (This includes all pregnancy outcomes such as live birth, currently pregnant, abortion, or miscarriage.)

- Yes
- No
- Unknown

Survey on fertility procedures and outcomes for patients treated with CAR-T cell therapy on behalf of the EBMT CTIWP

34. How many patients who attempted to conceive after CAR-T cell therapy successfully achieved pregnancy (please include all pregnancy outcomes such as live birth, ongoing pregnancy, abortion, or miscarriage)?

(Please enter 0 if no patients have conceived.)

Total number of patients who conceived:

[ ]

35. Among the patients who achieved pregnancy after CAR-T cell therapy, what was the earliest time point that a pregnancy occurred?

- ◯ 0-6 months
- ◯ 6-12 months
- ◯ 1-2 years
- ◯ 2-5 years
- ◯ >5 years

36. Among patients who conceived after CAR-T cell therapy, please indicate: Please fill all boxes and enter 0 if no patients.
Please check that the total of the numbers at the top of this page corresponds to the numbers in this question!

Number of patients who achieved a live birth:

Number of patients currently pregnant:

Number of patients with elective abortion:

Total number of patients who experienced an unsuccessful pregnancy outcome (e.g. premature delivery, miscarriage):

Number of patients who experienced premature delivery:

Number of patients who experienced a miscarriage:

Other reason for an unsuccessful pregnancy outcome, please specify and indicate number of patients:

Survey on fertility procedures and outcomes for patients treated with CAR-T cell therapy on behalf of the EBMT CTIWP

37. How many patients who attempted but failed to conceive or father children post-CAR-T cell therapy? (Please enter 0 if no patients have failed to conceived.)
Total number of patients who failed to conceive:

[ ]

38. Among the patients who attempted to conceive after CAR-T cell therapy, how many experienced a cytokine release syndrome (CRS)* grade of 3-4?

***ASTCT Consensus Grading scale (Lee 2019)**

Please provide the number of patients for each category:
(Please enter 0 if no patients.)

Among the patients who achieved a live birth:	
Among the patients currently pregnant:	
Among the patients with elective abortion:	
Among the patients who experienced premature delivery:	
Among the patients who experienced a miscarriage:	
Among the patients who attempted but failed to conceive:	

39. Among the patients who attempted to conceive after CAR-T cell therapy, how many had an Immune Effector Cell-Associated Neurotoxicity Syndrome (ICANS) (any grade) after CAR-T cell therapy?
***ASTCT Consensus Grading scale (Lee 2019)**

Please provide the number of patients for each category:
(Please enter 0 if no patients.)

Among the patients who achieved a live birth:	
Among the patients currently pregnant:	
Among the patients who experienced an abortion:	
Among the patients who experienced premature delivery:	
Among the patients who experienced a miscarriage:	
Among the patients who attempted but failed to conceive:	

40. On average, for patients who attempted but failed to conceive, how long have they been trying to conceive after CAR-T cell therapy?

- ◯ Less than 1 year
- ◯ 1 - < 3 years
- ◯ 3 - < 5 years
- ◯ 5 - < 7 years
- ◯ 7 years or more
- ◯ Unknown

41. How many patients started a family through surrogacy after unsuccessful attempts to conceive?
(Please enter 0 if no patients.)

Number of patients:

42. How many patients started a family through adoption after unsuccessful attempts to conceive?
(Please enter 0 if no patients.)

Number of patients:

[ ]

Survey on fertility procedures and outcomes for patients treated with CAR-T cell therapy on behalf of the EBMT CTIWP

Fertility procedures

43. Do you routinely monitor biological markers of fertility (i.e. testosterone, follicle-Stimulating Hormone (FSH))?

- ◯ Always
- ◯ Often
- ◯ Sometimes
- ◯ Never

Survey on fertility procedures and outcomes for patients treated with CAR-T cell therapy on behalf of the EBMT CTIWP

44. If you routinely monitor biological markers of fertility, which marker do you monitor in <u>women</u>? (Select all that apply)

- [ ] Follicle-Stimulating Hormone (FSH)
- [ ] Luteinizing Hormone (LH)
- [ ] Estradiol (E2)
- [ ] Anti-Müllerian Hormone (AMH)
- [ ] Progesterone
- [ ] Other (please specify)

45. If you routinely monitor biological markers of fertility, which marker do you monitor in <u>men</u>? (Select all that apply)

- [ ] Follicle-Stimulating Hormone (FSH)
- [ ] Luteinizing Hormone (LH)
- [ ] Estradiol (E2)
- [ ] Testosterone
- [ ] Inhibin B
- [ ] Other (please specify)

Survey on fertility procedures and outcomes for patients treated with CAR-T cell therapy on behalf of the EBMT CTIWP

46. Do you recommend within your center procedure for ovarian functional rest during CAR-T cell therapy?

- Always
- Often
- Sometimes
- Never

Survey on fertility procedures and outcomes for patients treated with CAR-T cell therapy on behalf of the EBMT CTIWP

47. What is your procedure(s) for ovarian functional rest during CAR-T cell therapy? (Select all that apply)

- ☐ Gonadotropin-releasing hormone (GnRH) agonists
- ☐ Progestin
- ☐ None
- ☐ Other (please specify)

48. If you recommend procedure(s) for ovarian functional rest during CAR-T cell therapy, at which point in time did you do so?

- ◯ Only during the aplastic phase
- ◯ Long-term ovarian functional resting procedure
- ◯ Other (please specify)

Survey on fertility procedures and outcomes for patients treated with CAR-T cell therapy on behalf of the EBMT CTIWP
Comments

49. Would you be interested to participate in the future in a retrospective study regarding fertility procedures and outcomes for patients treated with CAR-T cell therapy?

- ◯ Yes
- ◯ Maybe
- ◯ No, please specify reason:

50. Please let us know if you have any comments regarding this survey:
