# Supplementary material for: Fertility management and outcomes after CAR T-cell therapy: an international survey from the Cellular Therapy and Immunobiology working party of the European Society for Blood and Marrow Transplantation
Source: eClinicalMedicine. 2026 Jun 11;96:104014. doi: 10.1016/j.eclinm.2026.104014 (PMC13272563; doi:10.1016/j.eclinm.2026.104014)
Supplement: Supplementary Table S2 [file mmc2.pdf]

| Patients | Treatments before CAR T-Cells | Lymphodepletion used |
|---|---|---|
| **F 1** | First Line: FRALLE 2000 A (Arm A1); Second line: IntReALL 2010 (Arm A SR); Bridge to CAR T: Vincristine plus 6-mercaptopurine | Flu 30 mg/m2 x 3 days + Cy 500 mg/m2 x 2 days |
| **F 2** | First line: R-CHOP (×6 cycles) plus CNS prophylaxis with methotrexate; Second line: R-GDP (×5 cycles) –Bridging therapy: pembrolizumab (×1 cycle) | Flu 30 mg/m2 x 3 days + Cy 500 mg/m2 x 2 days |
| **F 3** | NA | NA |
| **F 4** | First line: R-DA-EPOCH –Second line: ICE | Flu 30 mg/m2 x 3 days + Cy 300 mg/m2 x 2 days |
| **F 5** | First line: R-CHOP (×6 cycles); Second line: R-DHAP (×2 cycles); Bridging to CAR T: mediastinal radiotherapy 30 Gy total dose. | Flu 30 mg/m2 x 3 days + Cy 500 mg/m2 x 2 days |
| **F 6** | First line: R-CHOP (×6 cycles); Second line: GDP (×2 cycles) | Flu 24 mg/m2 x 3 days + Cy 500 mg/m2 x 2 days |
| **F 7** | First line: R-CHOP (×6 cycles); Second line: Bendamustine ×6 cycles; Third line: Third line: ESHAP ×4 cycles followed by ASCT; Fourth line: Idelalisib + Lenalidomide; Fifth line: GIFOX ×2 cycles followed by DHAOx ; Sixth Line: Sixth line: Pixantrone | Flu 24 mg/m2 x 3 days + Cy 500 mg/m2 x 2 days |
| **F 8** | 1) First line: VCD followed by single ASCT (approximately 6.5 years prior to CAR-T); 2) Bridging therapy: Dara-Rd (number of cycles not documented) prior to CAR-T | Flu 30 mg/m2 x 3 days + Cy 300 mg/m2 x 2 days |
| **F 9** | Multiple lines of immuno-suppressive therapy including corticosteroids, Immuno-globulin, methotrexate, azathioprine, mycophenolate mofetil, and rituximab. | Flu 25 mg/m$^2$x 3 days + Cy 250 mg/m$^2$ x 3 days |
| **F 10** | Frist Line: R-CHOP (x 6cycles); Second Line: NA; Third Line: NA | Flu 30 mg/m2 x 3 days + Cy 500 mg/m2 x 2 days |
| **F 11** | Frist Line: R-CHOP (x 6cycles); Second Line: NA; Third Line: NA | Flu 30 mg/m2 x 3 days + Cy 500 mg/m2 x 2 days |
| **F 12** | Frist Line: R-CHOP (x 6cycles); Second Line: NA | Flu 30 mg/m2 x 3 days + Cy 500 mg/m2 x 2 days |
| **F 13** | First line: R-CHOP (×6 cycles); Bridging therapy: Pola-R (x 1 cycle) | Flu 30 mg/m2 x 3 days + Cy 500 mg/m2 x 2 days |
| **M 1** | First line: R-CHOP–Second line: ICE | Flu 30 mg/m2 x 3 days + Cy 300 mg/m2 x 2 days |
| **M 2** | First line: BCNU (×2 cycles) –followed by ASCT; Second line: Rituximab–Methotrexate–Ibrutinib (×2 cycles) | Flu 30 mg/m2 x 3 days + Cy 300 mg/m2 x 2 days |
| **M 3** | Induction 1: Daunorubicine-Asparaginase-Vincristine-Steroids, 2nd line: Induction 2: FLAG-Ida, Consolidation: Methotrexate-Asparaginase, TBF alloHSCT, 3rd line: FLAG-Ida, 4th line: Blinatumomab, + alloHSCT, DLI, 5th line: inotuzumab | Flu 30 mg/m2 x 3 days + Cy 300 mg/m2 x 2 days |
| **M 4** | Induction 1: Daunorubicine - Asparaginase - Vincristine- Steroids; Consolidation 1: Methotrexate, Etoposide, Cytarabine, Mercaptopurine; Consolidation 2: Steroids, daunorubicine, vincristine, cyclophosphamide, asparaginase; Maintenance and reinductions: mercaptopurine, methotrexate, vincristine, steroids, asparaginase. 2nd Line: induction 1: daunorubine, steroids, vincristine, asparaginase, rituximab + alloHSCT, 3rd line blinatumomab | Flu 30 mg/m2 x 3 days + Cy 300 mg/m2 x 2 days |
| **M 5** | Fist Line: R-CHOP; second line: R-DHAX + auto-HCT; Bridging to CAR T: R-ICE | Flu 30 mg/m2 x 3 days + Cy 500 mg/m2 x 2 days |
| **M 6** | 1) First Line: AllTogether High-risk 2) Second line: High-dose Methotrexate | Flu 30 mg/m2 x 3 days + Cy 500 mg/m2 x 2 days |

**Abbreviations**: CAR: chimeric antigen receptor; F: female; M: male; HCT: hematopoietic cell transplantation; auto: autologous; allo: allogeneic; FRALLE 2000 A (Arm A1) Induction: Prednisone 60 mg/m$^2$ continuously (days 1–28), vincristine 1.5 mg/m$^2$ on days 1, 8, 15, 22, daunorubicin 25–30 mg/m$^2$ on days 1, 8, 15, 22, L-asparaginase 5,000–10,000 IU/m$^2$ administered multiple times during induction; Consolidation: Cyclophosphamide 1,000 mg/m$^2$ (single or repeated blocks), cytarabine 75 mg/m$^2$ for several consecutive days, 6-mercaptopurine 60 mg/m$^2$/day; Delayed intensification: Vincristine 1.5 mg/m$^2$, dexamethasone 6–10 mg/m$^2$/day, anthracycline (e.g., doxorubicin 25 mg/m$^2$), cyclophosphamide 1,000 mg/m$^2$, cytarabine, thiopurine (6-mercaptopurine or thioguanine); Maintenance: 6-mercaptopurine 50–75 mg/m$^2$/day continuously, methotrexate 20–25 mg/m$^2$ weekly, vincristine 1.5 mg/m$^2$ and steroid pulses; IntReALL 2010 (Arm A, SR) Reinduction: Dexamethasone 10 mg/m$^2$/day, vincristine 1.5 mg/m$^2$ weekly, PEG-asparaginase 1,000–2,500 IU/m$^2$, anthracycline (idarubicin 10–12 mg/m$^2$ or equivalent); Consolidation: High-dose methotrexate 1–5 g/m$^2$ per cycle, cytarabine 1–3 g/m$^2$, cyclophosphamide 1,000 mg/m$^2$, etoposide (~100 mg/m$^2$/day for several days depending on block); Maintenance: 6-mercaptopurine 50–75 mg/m$^2$/day plus methotrexate 20–25 mg/m$^2$ weekly; Vincristine + 6-mercaptopurine: Vincristine 1.5 mg/m$^2$ weekly plus 6-mercaptopurine 25 mg/m$^2$/day; R-CHOP + CNS prophylaxis: Rituximab 375 mg/m$^2$ day 1, cyclophosphamide 750 mg/m$^2$ day 1, doxorubicin 50 mg/m$^2$ day 1, vincristine 1.4 mg/m$^2$ (max 2 mg) day 1, prednisone 100 mg/day days 1–5 (q21), methotrexate 3–3.5 g/m$^2$ IV or 12–15 mg intrathecal for CNS prophylaxis; R-GDP: Rituximab 375 mg/m$^2$ day 1, gemcitabine 1,000 mg/m$^2$ days 1 and 8, dexamethasone 40 mg/day days 1–4, cisplatin 75 mg/m$^2$ day 1 (or 25 mg/m$^2$ days 1–3); Pembrolizumab: Pembrolizumab 200 mg IV day 1; R-DA-EPOCH: Rituximab 375 mg/m$^2$ day 1, etoposide 50 mg/m$^2$/day continuous infusion days 1–4, doxorubicin 10 mg/m$^2$/day continuous infusion days 1–4, vincristine 0.4 mg/m$^2$/day continuous infusion days 1–4, cyclophosphamide 750 mg/m$^2$ day 5, prednisone 60 mg/m$^2$/day days 1–5 (dose-adjusted); ICE: Ifosfamide 5 g/m$^2$ day 2, carboplatin AUC 5 day 2, etoposide 100 mg/m$^2$ days 1–3; R-DHAP (×2 cycles): Rituximab 375 mg/m$^2$ day 1, dexamethasone 40 mg/day days 1–4, cisplatin 100 mg/m$^2$ continuous infusion day 1, cytarabine 2 g/m$^2$ every 12 hours on day 2; GDP: Gemcitabine 1,000 mg/m$^2$ days 1 and 8, dexamethasone 40 mg/day days 1–4, cisplatin 75 mg/m$^2$ day 1 (or 25 mg/m$^2$ days 1–3); Bendamustine (×6 cycles): Bendamustine 90 mg/m$^2$ days 1–2 (q28), rituximab omitted; ESHAP: Etoposide 40 mg/m$^2$ days 1–4, methylprednisolone 500 mg/day days 1–5, cytarabine 2 g/m$^2$ day 5, cisplatin 25 mg/m$^2$ days 1–4; GIFOX followed by DHAOx: Gemcitabine 1,000 mg/m$^2$ days 1 and 8, ifosfamide 1,500 mg/m$^2$ days 1–3, oxaliplatin 100 mg/m$^2$ day 1, dexamethasone 40 mg/day days 1–4, cytarabine 2 g/m$^2$ day 2; Pixantrone: Pixantrone 50 mg/m$^2$ days 1, 8, 15 (q28); VCD: Bortezomib 1.3 mg/m$^2$ (days 1, 4, 8, 11), cyclophosphamide 300–500 mg/m$^2$ (days 1, 8, 15), dexamethasone 20–40 mg (days 1–2, 4–5, 8–9, 11–12); Pola-R: Polatuzumab vedotin 1.8 mg/kg day 1, rituximab 375 mg/m$^2$ day 1; BCNU-based regimen: Rituximab 375 mg/m$^2$ day 1, methotrexate 3–3.5 g/m$^2$ day 1, cytarabine 2 g/m$^2$ every 12 hours days 2–3, carmustine 100–150 mg/m$^2$ day 4; Rituximab–Methotrexate–Ibrutinib (×2 cycles): Rituximab 375 mg/m$^2$ day 1, methotrexate 3–3.5 g/m$^2$ day 1, ibrutinib orally continuously; FLAG-Ida: Fludarabine 30 mg/m$^2$ IV days 1–5, cytarabine 2 g/m$^2$ IV days 1–5, G-CSF 5 mcg/kg SC from day 0/1, idarubicin 10 mg/m$^2$ IV days 1–3; AllTogether High-Risk Induction: Dexamethasone 6 mg/m$^2$ continuously days 1–28, vincristine 1.5 mg/m$^2$ days 1, 8, 15, 22, daunorubicin 25 mg/m$^2$ days 1, 8, 15, 22, PEG-asparaginase 1,500/1,000 IU/m$^2$ days 4 and 18, CNS involvement at diagnosis treated with methotrexate 12 mg intrathecal initially followed by triple intrathecal therapy ×9 (methotrexate 12 mg, cytarabine 30 mg, methylprednisolone 16 mg); AllTogether High-Risk Consolidation: Dexamethasone taper, vincristine 1.5 mg/m$^2$ day 29, PEG-asparaginase 1,500/1,000 IU/m$^2$ days 32, 46, 60, cyclophosphamide 1,000 mg/m$^2$ days 36 and 50, cytarabine 75 mg/m$^2$ for 4 days starting on days 38, 45, 52, 59, 6-mercaptopurine 60 mg/m$^2$/day from days 36–63; Flu: Fludarabine; Cy, Cyclophosphamide
