# Supplementary material for: Fertility management and outcomes after CAR T-cell therapy: an international survey from the Cellular Therapy and Immunobiology working party of the European Society for Blood and Marrow Transplantation
Source: eClinicalMedicine. 2026 Jun 11;96:104014. doi: 10.1016/j.eclinm.2026.104014 (PMC13272563; doi:10.1016/j.eclinm.2026.104014)
Supplement: Translated Abstracts [file mmc3.pdf]

The following translations in French were submitted by the authors and we reproduce them as supplied. They have not been peer reviewed. Our editorial processes have only been applied to the original abstract in English, which should serve as reference for this manuscript.

Abstract:

La thérapie par cellules CAR-T est devenue un traitement efficace pour les maladies du sang et, plus récemment, pour certaines maladies auto-immunes. Avec son utilisation croissante, il est essentiel de mieux comprendre ses effets à long terme, notamment son impact sur la fertilité, un domaine encore peu étudié et sans recommandations officielles.

Nous avons mené une enquête européenne au nom de l'EBMT, auprès de 247 centres spécialisés. 40% des centres ont répondu. Au total, 24 grossesses ont été rapportées chez 19 patients: 18 naissances vivantes, 2 grossesses en cours et 4 fausses couches. Dix-huit grossesses concernaient des femmes traitées par CAR-T, six étaient des partenaires d'hommes traités. La majorité des grossesses chez les femmes (83%) est survenue naturellement, La plupart des centres proposent un conseil en fertilité et des procédures de préservation avant le traitement, bien qu'avec des différences importantes entre les centres.

Il s'agit de la plus grande série grossesses après CAR-T, la première en Europe. Avec une utilisation de plus en plus précoce de cette thérapie chez des patients jeunes, il est urgent d'intégrer des stratégies standardisées de conseil et de préservation de la fertilité dans la pratique clinique.

The following translations in Italian were submitted by the authors and we reproduce them as supplied. They have not been peer reviewed. Our editorial processes have only been applied to the original abstract in English, which should serve as reference for this manuscript.

Abstract:

La terapia con cellule CAR-T è diventata un trattamento efficace per le malattie del sangue e, più recentemente, per alcune malattie autoimmuni. Con l'aumentare del suo utilizzo, è fondamentale comprendere gli effetti a lungo termine, tra cui l'impatto sulla fertilità, un tema ancora poco studiato e privo di linee guida specifiche.

Abbiamo condotto un'indagine europea, per conto dell'EBMT, coinvolgendo 247 centri specializzati. Il 40% dei centri ha risposto. Sono state riportate 24 gravidanze in 19 pazienti: 18 nascite, 2 gravidanze in corso e 4 aborti spontanei. Diciotto gravidanze riguardavano donne trattate con CAR-T, sei erano partner di uomini trattati. La maggior parte delle gravidanze nelle donne (83%) è avvenuta naturalmente. La maggior parte dei centri offre consulenza sulla fertilità e procedure di preservazione prima del trattamento, anche se con importanti differenze tra centri.

Questo è il più grande casistica riportata di gravidanze dopo CAR-T, la prima in Europa. Con l'utilizzo sempre più precoce di questa terapia in pazienti giovani, è urgente integrare strategie standardizzate di counselling e preservazione della fertilità nella pratica clinica.

The following translations in Spanish were submitted by the authors and we reproduce them as supplied. They have not been peer reviewed. Our editorial processes have only been applied to the original abstract in English, which should serve as reference for this manuscript.

Abstract:

La terapia con células CAR-T se ha consolidado como un tratamiento eficaz para las enfermedades hematológicas y, más recientemente, para determinadas enfermedades autoinmunes. A medida que su uso clínico incrementa, resulta fundamental comprender los efectos a largo plazo, incluido el impacto sobre la fertilidad, un área aún poco estudiada y sin guías clínicas específicas.

Se realizó una encuesta europea en nombre de EBMT, en la que participaron 247 centros especializados. Respondió el 40% de los centros. Se reportaron 24 embarazos en 19 pacientes: 18 nacidos vivos, 2 embarazos en curso y 4 abortos espontáneos. Dieciocho embarazos correspondieron a mujeres tratadas con CAR-T, mientras que seis se produjeron en las parejas de varones tratados. La mayoría de los embarazos en mujeres (83%) ocurrieron de forma natural. La mayoría de los centros ofrece asesoramiento sobre fertilidad y procedimientos de preservación antes del tratamiento, aunque existen diferencias relevantes entre centros.

Esta es la mayor serie de casos de embarazos tras terapia CAR-T y la primera reportada en Europa. Dado el uso cada vez más precoz y en pacientes jóvenes, de esta terapia, es urgente integrar estrategias estandarizadas de asesoramiento y preservación de la fertilidad en la práctica clínica.
